# Next-Generation Sequencing Gene Panels in Inheritable Cardiomyopathies and Channelopathies: Prevalence of Pathogenic Variants and Variants of Unknown Significance in Uncommon Genes

**DOI:** 10.3390/biom12101417

**Published:** 2022-10-03

**Authors:** Cristina Mazzaccara, Raffaella Lombardi, Bruno Mirra, Ferdinando Barretta, Maria Valeria Esposito, Fabiana Uomo, Martina Caiazza, Emanuele Monda, Maria Angela Losi, Giuseppe Limongelli, Valeria D’Argenio, Giulia Frisso

**Affiliations:** 1 Department of Molecular Medicine and Medical Biotechnologies, University of Naples Federico II, 80131 Napoli, Italy; 2 CEINGE Biotecnologie Avanzate, 80145 Napoli, Italy; 3 Department of Advanced Biomedical Sciences, University of Naples Federico II, 80131 Napoli, Italy; 4 Department of Medicine, Division of Cardiology, University of Colorado Anschutz Medical Campus, Aurora, CO 80045, USA; 5 Monaldi Hospital, AO Colli, 80131 Napoli, Italy; 6 Department of Translational Medical Sciences, University of Campania ‘Luigi Vanvitelli’, 81100 Caserta, Italy; 7 Department of Human Sciences and Quality of Life Promotion, San Raffaele Open University, 00166 Roma, Italy

**Keywords:** cardiomyopathies, channelopathies, next-generation sequencing, genetic testing, uncommon genes, diagnostic sensitivity, genes panel analysis, inherited diseases

## Abstract

The diffusion of next-generation sequencing (NGS)-based approaches allows for the identification of pathogenic mutations of cardiomyopathies and channelopathies in more than 200 different genes. Since genes considered uncommon for a clinical phenotype are also now included in molecular testing, the detection rate of disease-causing variants has increased. Here, we report the prevalence of genetic variants detected by using a NGS custom panel in a cohort of 133 patients with inherited cardiomyopathies (n = 77) or channelopathies (n = 56). We identified 82 variants, of which 50 (61%) were identified in genes without a strong or definitive evidence of disease association according to the NIH-funded Clinical Genome Resource (ClinGen; “uncommon genes”). Among these, 35 (70%) were variants of unknown significance (VUSs), 13 (26%) were pathogenic (P) or likely pathogenic (LP) mutations, and 2 (4%) benign (B) or likely benign (LB) variants according to American College of Medical Genetics (ACMG) classifications. These data reinforce the need for the screening of uncommon genes in order to increase the diagnostic sensitivity of the genetic testing of inherited cardiomyopathies and channelopathies by allowing for the identification of mutations in genes that are not usually explored due to a currently poor association with the clinical phenotype.

## 1. Introduction

Inheritable cardiomyopathies and channelopathies are disorders with phenotypic and genetic heterogeneous features caused by the presence of structural or electrical heart abnormalities [1]. Variable penetrance and incomplete expression are also common and may be due to the interaction of the causal mutation with modifier genes, epigenetic changes, environmental factors, or individual factors such as age, gender, ethnicity, or physical activity [2]. According to their functional and morphological features, cardiomyopathies are commonly classified as hypertrophic cardiomyopathy (HCM), arrhythmogenic cardiomyopathy (ACM), dilated cardiomyopathy (DCM), or restrictive cardiomyopathy (RCM) [3]. Channelopathies are arrhythmic disorders in patients without structural heart abnormalities that are usually due to genetic alterations in genes coding for cardiac ion channels or their associated proteins. The main channelopathies are: Brugada syndrome (BrS), long QT syndrome (LQTS), short QT syndrome (SQTS), and catecholaminergic polymorphic ventricular tachycardia (CPVT) [4].

Both cardiomyopathies and channelopathies can cause sudden cardiac death (SCD), and this tragic event may represent the onset of an inherited heart disease in asymptomatic individuals, including subjects who practice intense physical activity, such as elite athletes [5,6,7,8,9].

Over the last two decades, the knowledge of the molecular bases of cardiomyopathies and channelopathies has gradually increased, and putatively associated variants have been identified in more than 200 genes [10,11,12,13,14,15,16,17,18,19,20,21,22]. Indeed, while some genes are strongly related to a clinical phenotype and are highly penetrant causative genes, others have only rarely been identified in affected subjects, and the association with the disease seems to be poor. Currently, mutations in about 100 different genes are associated to HCM [5,23]. Nevertheless, eight main genes, *MYBPC3*, *MYH7*, *TNNT2*, *TNNI3*, *TPM1*, *ACTC1*, *MYL2,* and *MYL3,* account for up to 65–70% of all HCM cases [24,25,26], while other uncommon genes seem to be globally involved in about 10% of HCM cases [12,27,28,29,30]. Similarly, 51 curated genes were associated with idiopathic DCM [31], explaining up to 40–50% of DCM cases [24]. Among these, mutations in *TTN* gene account for up to 20–25% of DCM cases, while mutations occurring in other genes have rarely been identified [32,33,34,35,36,37,38,39,40,41,42]. With regard to ACM, pathogenic variants in each of the main cardiac desmosome genes were identified in more than 50% of the affected subjects [24,43,44], a smaller fraction carrying pathogenic mutations in nondesmosomal genes [43,45,46]. Lastly, a combination of sarcomeric and cytoskeletal genes were detected in half of RCM patients [47]. 

Similar considerations also apply to channelopathies. About 20–25% of BrS patients carry a mutation in the *SCN5A* gene, while other disease-related genes explain an additional 5% of all BrS cases [5,48,49,50]. With regard to LQTS, mutations in the *KCNQ1, KCNH2,* and *SCN5A* genes are found in about 75% of patients, while 5–10% of cases are due to genetic mutations in uncommon genes. The main gene associated with SQTS is *KCNH2,* reported in about 15% of SQTS patients; other uncommon genes were also reported [51]. Lastly, genes mainly associated with CPVT are *RYR2* (60%) and *CASQ2* (5%); other genes are rarely reported (≤1% each) [52]. Supplementary Table S1 shows the genes reported in the literature associated with the above mentioned cardiomyopathies and channelopathies [26].

Most previously reported genes are commonly included in diagnostic tests. Moreover, in recent years, advances in high-throughput sequencing strategies, such as next-generation sequencing (NGS), have revolutionized the diagnosis of inherited heart diseases in terms of expanding the number of involved genes and the discovery of new genes potentially associated with the diseases [5,53,54,55,56,57]. 

This wide genetic scenario highlights the need for a standardized method to estimate the genetic evidence and the clinical validity of gene–disease relationships. A proposed framework to evaluate relevant genetic and experimental evidence supporting or contradicting a gene–disease relationship is represented by the NIH-funded Clinical Genome Resource (ClinGen) (https://search.clinicalgenome.org/, accessed on 26 September 2022) [58,59], a standardized evidence-based framework, from expert panel curation. ClinGen is an authoritative open-access online resource that defines genes and variants on the basis of the level of evidence of disease association, which can be strong, definitive, or moderate. ClinGen also reports genes that have a gene–disease association in the literature data, but present limited or conflicting evidence to support a causal role in the disease since the time of the initial report. These genes are classified as limited, disputed, or refuted.

In this manuscript, we report the prevalence and type of genetic variants identified in patients with cardiomyopathies and channelopathies, focusing on genes with weaker evidence (limited, disputed, refuted) of disease association and genes not reported for the specific phenotype according to the ClinGen definition. Here, we define “uncommon genes” as all limited, disputed, or refuted genes, or those without a reported association with the clinical phenotype of the patients.

## 2. Materials and Methods

### 2.1. Patient Enrolment and DNA Extraction

In total, 133 unrelated patients with a clinical diagnosis or suspicion of an inherited cardiac disease (cardiomyopathy or channelopathy) were enrolled from June 2018 to date and followed up on an average period of 3 years at the Department of Cardiomyopathy and Inherited Heart Disease Clinic, UOC Cardiology, University of Campania ‘Luigi Vanvitelli’ of Naples, and at the Center for the Study of Hypertrophic Cardiomyopathies of the Cardiology Division of the Federico II University of Naples. Genetic testing was performed at the CEINGE diagnostic laboratory.

The reference clinicians of the patients required the molecular analysis to investigate the presence of a possible genetic disease. A blood sample was collected in EDTA from each study subject. Each patient gave their written informed consent to molecular analysis according to the tenets of the Helsinki Declaration [60] and the internal ethics committee (N. 77/21). Genomic DNA was extracted using a Maxwell 16 instrument (Promega, Madison, WI, USA). DNA quality was assessed with a Nanodrop (Thermo Fisher, Waltham, MA, USA) spectrophotometer and Tape Station (Agilent, Santa Clara, CA, USA) analyzers to verify their concentration, purity, and integrity before proceeding to library preparation.

### 2.2. Molecular Analysis

Molecular testing was carried out by analyzing a panel of target genes through an NGS-based procedure. In particular, 2 custom panels were used: (1) a first-level panel including 60 diagnostic genes associated to the different genetic cardiomyopathies, including HCM, DCM, ACM, and channelopathies, such as LQTS and BrS; and (2) an enlarged panel of 129 genes including rarer genes; this panel was used in cases of complex or unclear phenotypes and in the presence of a positive family history for SCD. In both cases, HaloPlex technology (Agilent, Santa Clara, CA, USA) was used for library preparation. In detail, each genomic DNA sample was fragmented using a pool of restriction enzymes. The obtained fragments were enriched with hybridization with the custom capture probes, and then purified and PCR-amplified to obtain a DNA library or sample. During this procedure, each genomic DNA sample was univocally tagged with a barcode sequence to allow for sample multiplexing during the subsequent sequencing step.

The obtained enriched and indexed libraries were sequenced using the MiSeq (Illumina, San Diego, CA, USA) instrument (2 × 250 PE). Alissa software (Agilent, Santa Clara, CA, USA) was used to perform sequence data analysis. This tool allows for the alignment of the sequences to the reference genome to obtain a list of genomic variants that can be prioritized using a customizable pipeline in order to highlight pathogenic mutations or potentially pathogenic variants. This pipeline allows for variant classification based on their position with regard to the gene structure (exonic or intronic), population frequency, coding effect, ClinVar classification, and functional predictions. In addition, we classified the variants according to American College of Medical Genetics and Genomics (ACMG) guidelines adapted to cardiomyopathy [61]. In detail, all variants were evaluated for their frequency in international population database gnomAD (http://gnomad.broadinstitute.org/, accessed on 26 September 2022): we excluded variants showing minor frequency alleles (MAF) below the calculated maximal tolerated allele frequency for the specific cardiomyopathy [62]. According to these criteria, the maximal credible allele frequency was 0.000084 for DCM, 0.00004 for HCM, 0.000092 for ACM, 0.00001 for BrS, and 0.0000082 for LQTS [62]. However, we kept 10 variants showing an MAF greater than the threshold because they are reported as pathogenetic in the HGMD database. The latter classifies variants on the basis of the interpretation at first reporting; thus, even if it is less valuable with respect to ACMG classification and more prone to subsequent modifications, it may provide additional data supporting the variants’ correct classification and subsequent interpretation. Moreover, bioinformatic pathogenicity evaluation for missense variants was performed by using multiple in silico tools, including Grantham distance and an Align–GVGD matrix. The splicing module from Alamut visual v.2.7.0.0 software was used to test the pathogenicity of a possible splicing variant. In some cases, we had informative families, i.e., ones having at least 3 affected relatives in which we analyzed variant cosegregation. In families with one affected member being a noncarrier, the variant is considered to be non-disease-causing. Pathogenic or doubtful variants were confirmed with Sanger sequencing.

## 3. Results

In total, 133 independent subjects underwent genetic testing because of a clinical suspicion of inherited cardiomyopathy or channelopathy over a period of enrollment of 3 years (2018–2020). The average sequencing coverage of the target regions was in the range of 150–250X with a 50X minimal acceptable threshold. In detail, 60/133 patients showing a phenotype, and/or laboratory or instrumental data supporting a clinical diagnosis of a specific inherited cardiomyopathy according to international guidelines were analyzed using a first-level panel including 60 main diagnostic genes that had been associated with different cardiomyopathies/channelopathies. The remaining 73/133 subjects, showing unclear cardiological clinical signs and/or a positive family history for SCD, were analyzed using an enlarged 129 gene panel. In our cohort of 133 patients, we detected a total of 82 variants, excluding polymorphisms. The variants, shown in Table 1, were reported following Human Genome Variation Society (HGVS) nomenclature (http://www.HGVS.org/varnomen, accessed on 26 September 2022), and annotated according to the Human Gene Mutation Database (HGMD) Professional 2020.4, (http://www.hgmd.cf.ac.uk/, accessed on 26 September 2022), NCBI SNP Database (http://www.ncbi.nlm.nih.gov, accessed on 26 September 2022), and ClinVar database (https://www.ncbi.nlm.nih.gov/clinvar, accessed on 26 September 2022).

Among the 82 detected variants, 27 were novel as they were missing in the reference SNP, ClinVar, and HGMD databases. The ClinVar database reported 39/82 variants. Of these variants, 20 were classified as pathogenic or likely pathogenic (P/LP), 13 as conflicting (CI), and 6 of uncertain or unknown significance (VUS). The remaining 43 variants were not reported (NR) in ClinVar. HGMD reported 38/82 variants as pathogenic or likely pathogenic, while the remaining 44 were not reported. According to ACMG interpretation criteria, 39/82 variants were classified as P/LP (48%), of which 6 were novel (without reference SNP ID number), and 41/82 as VUSs (50%), of which 26 were novel ones. Furthermore, two of the identified variants (2%) were classified by ACMG guideline as benign or likely benign (B/LB). According to ACMG criteria, we globally identified P/LP variants in 53 patients (40% of the analyzed population), VUSs in 38 patients (29%), and B/LB variants in 4 patients (3%). In addition, 11 patients carried multiple variants. We did not identify any pathogenic mutations or VUSs in the remaining 38 patients (29%), who showed only known polymorphisms and/or LB variants in the analyzed genes. If we separately analyzed the two cohorts of patients, among the 60 patients admitted to the first-level test, 24 carried a P/LP variant (41%, 7 channelopathy and 17 cardiomyopathy patients) and 24 carried a VUS (41%, 7 channelopathy and 17 cardiomyopathy patients). Similarly, among the 73 patients analyzed using the enlarged panel, 29 carried a P/LP variant (40%, 4 channelopathy and 25 cardiomyopathy patients) and 14 carried a VUS (19%, 6 channelopathy and 8 cardiomyopathy patients).

**Figure 1:**
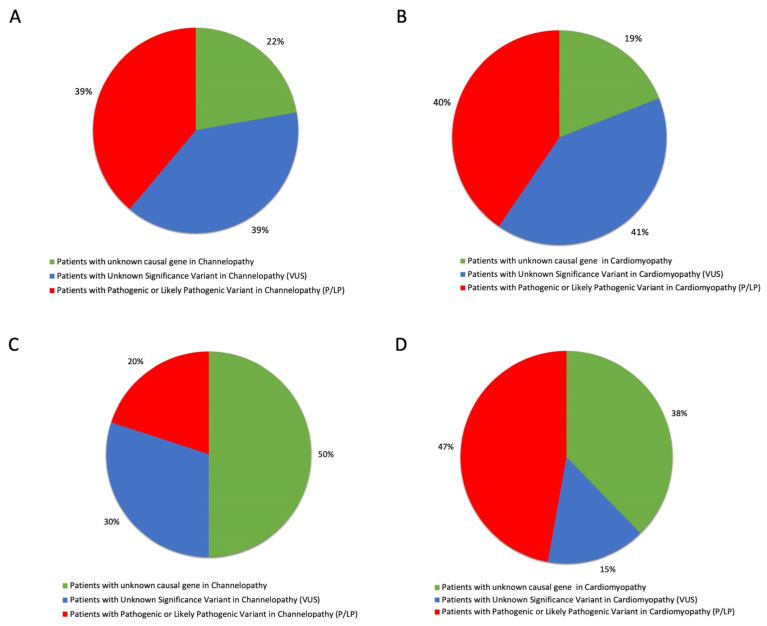
Percentage of channelopathy and cardiomyopathy patients carrying pathogenic/likely pathogenic (P/LP) and unknown significant (VUS) variants, as identified using (**A**,**B**) first-level and (**C**,**D**) extended panels. Variants were classified according to ACMG guideline classification with respect to patients without a known causative gene.

Figure 1 shows the distribution of P/LP and VUS variants among channelopathy (Figure 1A,C) and cardiomyopathy (Figure 1B,D) patients analyzed via the two genes panels. The percentage of patients with an unknown causal gene included subjects without pathogenic mutations or VUSs, carrying known polymorphisms and/or LB variants in the analyzed genes.

In total, 11 patients (8.3%) carried multiple variants: in particular, 3 patients carried two different P/LP mutations, 3 patients carried 1 P/LP mutation and a VUS, and 5 patients carried more than one VUS. We analyzed 5/11 patients with the first-level test, and 6/11 with the enlarged one. Furthermore, 7/11 patients showed double mutations in uncommon genes with respect to their phenotype, 2 patients were doubly mutated in main genes, and 2 patients showed one mutation in an uncommon and the other in a main gene.

Globally, among the 82 variants, 50 (61%) were localized in genes without reported evidence, or classified with limited or disputed evidence for a role in cardiomyopathies or channelopathies in ClinGen (uncommon genes), while 32 (39%) were had a definitive or moderate association with the disease. 

Among the 50 identified variants in uncommon genes, 48 were P/LP or VUS (13 as P/LP and 35 as VUSs) and 2 as B/LB according to ACMG classification. In addition, 11/13 P/LP and 14/35 VUSs were identified among patients who had undergone extended molecular analysis.

In particular, 9 ACM patients carried variants in the *CACNB2* (n = 2), *HCN4* (n = 1), *KCNE3* (n = 1), *LDB3* (n = 1), *MYH6* (n = 1), *PLN* (n = 1), *POLG* (n = 1), and *RYR2* (n = 1) genes. One ACM patient was a compound heterozygous (confirmed with segregation analysis) carrier of two missense variants (c.752C>T and c.1760C>T) in the *POLG* gene. Furthermore, c.40_42delAGA (p.Arg14del) in the *PLN* gene was present in both a patient with ACM and an independent DCM patient. ClinGen reports a definitive association between the *PLN* gene and the DCM but not ACM phenotype [63]. 

In addition, 17 independent HCM patients carried pathogenic variants or VUSs in the following uncommon genes: *ABCC9*, *ANK2*, *CAV3, CTNNA3*, *DSP*, *KCNQ1, FHL1, OBSCN*, *PKP2*, *RYR2, TGFB3* (reported in one patient each); *RAF1* (n = 2), *SCN10A* (n = 2), and *TTN* (n = 2). 

In total, 14 independent BrS patients carried P/LP or VUSs in the following uncommon genes: *ANK2*, *CACNA1D, CACNA2D1*, *CASQ2* (n = 2), *CAV3*, *DSC2*, *KCNE3*, *KRAS*, *MYBPC3*, *POLG*, *PRDM16*, *SGCD*, and *TGFB3*.

In 3 DCM patients, we identified 3 VUSs in uncommon genes *KCNQ1, TBX1,* and *TRPM4*. Lastly, in LQTS patients, we identified 3 VUSs in uncommon genes *KRT17, LAMA3,* and *POLG*. 

The detailed distribution of all identified variants divided by clinical phenotype is reported in Table 1.

**Figure 2:**
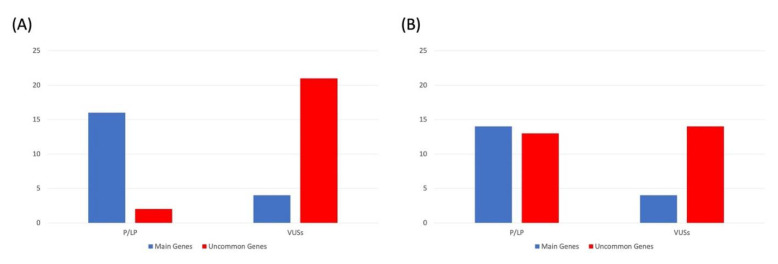
Number of pathogenic/likely pathogenic (P/LP) variants and VUSs according to ACMG guidelines, identified in the patients analyzed using (**A**) first-level and (**B**) enlarged panels and divided by main and uncommon genes.

Overall, in this study group, we detected 39 pathogenic or likely pathogenic mutations, of which 26 were identified in main genes (67%) and 13 (33%) in uncommon genes. Furthermore, we identified 41 VUSs, of which 35 (85%) in uncommon genes and 6 (15%) in main genes. Our findings indicate that P/LP variants are more likely to be identified in main genes, while VUSs are mostly identified in uncommon genes. When considering the two analyzed cohorts separately, we found that the number of variants (both P/LP and VUSs) in the main genes was almost the same within the two different panels; the number of P/LP variants identified in uncommon genes increased when the enlarged panel was used (Figure 2).

**Figure 3:**
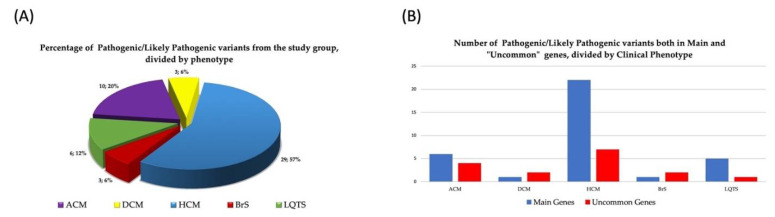
(**A**) Percentage and number of pathogenic or likely pathogenic variants collectively identified in the study cohort and (**B**) divided by main and uncommon genes according to clinical phenotype.

As shown in Figure 3A, P/LP mutations in both main and uncommon genes (accordingly with association with the reported cardiomyopathy in Clinical Genome Resource) were mainly detected in HCM patients (57% of the P/LP variants), followed by ACM (20%), LQTS, DCM, and BrS patients (12%, 6% and 6%, respectively). Among ACM, HCM, BrS, DCM, and LQTS patients carrying P or LP mutations, 4/10 (40%), 7/29 (24%), 2/3 (67%), 2/3 (67%), 1/6 (17%) were detected in uncommon genes, respectively (Figure 3B). Only 3 (19%) of these variants were identified in patients who had undergone the first-level test, while the remaining 13 (81%) were identified with the enlarged-gene panel.

**Figure 4:**
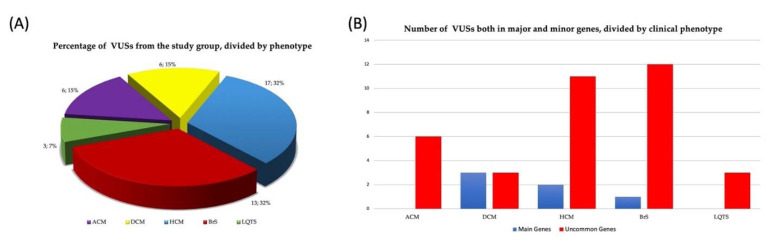
(**A**) Percentage and number of VUSs collectively identified in the study cohort and (**B**) divided by main and uncommon genes according to clinical phenotype.

Furthermore, among the 41 identified VUSs, 6 (15%) were detected in ACM, 6 (15%) in DCM, 13 (32%) in HCM, 13 (32%) in BrS, and 3 (7%) in LQTS patients (Figure 4A). Interestingly, 35/41 VUSs (85%) fell into the uncommon-gene group, with 22 (63%) identified in patients who had undergone the first-level test and 13 (37%) in those who performed the enlarged one. When we analyzed the distribution of the VUSs in uncommon and main genes per each disease, we found a higher percentage of VUSs in common genes in all the diseases with the exception of DCM (Figure 4B).

**Figure 5:**
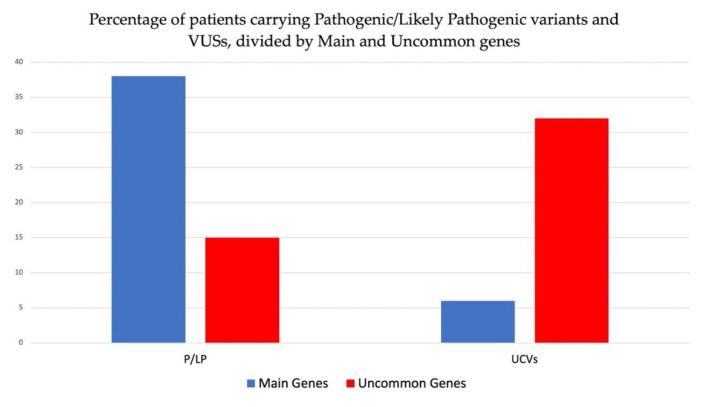
Percentage of patients carrying pathogenic or likely pathogenic variants and VUSs collectively identified in study cohort and divided by main and uncommon genes.

In the enrolled patients among 133 analyzed subjects, we detected 53 patients 40%) carrying at least one P or LP variants. In particular, 15 out of 53 patients (28%) carried P or LP variants in uncommon genes and 38 (72%) in main genes (Figure 5). Table 2 shows the demographic, clinical, and instrumental data of eight patients carrying pathogenic or likely pathogenic variants detected in uncommon genes with respect to the clinical diagnosis or suspicion for the inherited cardiac disease (cardiomyopathy or channelopathy). All except 1 patient (NA04) were analyzed with the enlarged panel of 129 genes, including rarer genes.

These eight patients were recruited according to the physician’s clinical suspicion for an inherited cardiomyopathy or channelopathy. In detail, four HCM patients carried mutations in uncommon genes *ABCC9, KCNQ1*, *PKP2* and *RAF1*, and two BrS patients in uncommon genes *MYBPC3* and *POLG*. Furthermore, we identified one ACM patient carrying a mutation in uncommon gene *KCNE3* and one ACM patient *in trans* carrying two variants in uncommon gene *POLG* (c.752C>T and c.1760C>T), classified as LP/P according to the novel ACMG criteria. 

Furthermore, VUSs were identified in 38 out of 133 patients (29%), of which 32 (84%) showed VUSs in uncommon genes and 6 (16%) in main genes (Figure 5). 

## 4. Discussion

In this study, we report the results of a targeted-NGS-based genetic screening carried out on 133 unrelated patients from South Italy diagnosed with cardiomyopathy or channelopathy. We used a large panel of 129 cardiomyopathy-related genes to increase the yield of genetic testing considering the genetic heterogeneity and variable and overlapping phenotypes, which is a characteristic of hereditary structural and electric cardiac diseases. Using the clinical genome resource approach to assess the strength of gene–disease association, we focused on variants in genes without definitive or strong evidence for an association with cardiomyopathies or channelopathies, which we defined as uncommon causal genes. 

Globally, our NGS panel test demonstrated a yield of 40% if we considered the genetic test to be positive in the presence of pathogenic or likely pathogenic variants in both main and uncommon genes. When we included the VUSs, the global yield of the test increased up to 69%. 

We detected 39 pathogenic or likely pathogenic variants according to the ACMG classification in 53 patients (44 cardiomyopathies and 9 channelopathies), including 13 patients carrying P or LP variants in uncommon genes. The role of these uncommon genes as causal genes should be evaluated in the context of the clinical manifestations and the evidence of the functional effects of the variant on the encoded protein; moreover, when large pedigrees are available, the analysis of the segregation of the genetic variant with the phenotype allows for confirming the phenotype–genotype association. Furthermore, we verified whether similar gene–disease associations to those identified in our study were reported in the literature. For example, in our study cohort, we detected a mutation in sarcomeric gene *MYBPC3* (c.906-1G>C; rs587776700) in a BrS patient. Experimental studies showed that this variant disrupts mRNA splicing, leading to a loss of protein function [64]. Some studies reported a *MYBPC3* mutation in patients showing the Brugada phenotype [65,66]. In addition, the literature reported cases of Brugada phenocopies in patients carrying a pathogenic mutation in the *MYBPC3* gene [67], speculating that Brugada type 1 ECG could be an early sign of an occult structural heart disease. Brugada syndrome is generally considered to be a disease of the ion channels. However, recent studies have also identified structural heart impairment, i.e., epicardial surface and interstitial fibrosis, increased collagen, and reduced contractility in BrS patients. These observations agree with the finding of causal mutations in genes that are molecular causes of cardiomyopathies, such as sarcomeric genes [66]. These data suggest that structural and electrical cardiac alterations may be present in BrS patients, which may develop from the same molecular alteration.

Similarly, although HCM is commonly considered to be a disease of the sarcomere, recent literature data reported an association of the HCM phenotype with mutations in the *ABCC9* [48,68], *PKP2* [69,70,71], and *RAF1* [72] genes, and in other nonsarcomeric genes [73]. The pathogenic mechanisms leading to the development of the HCM phenotype need to be elucidated.

Furthermore, in our study, we identified (L)P variants in the *POLG* gene in two patients with BrS and ACM, respectively. Our data are in accordance with recently published data in which the *POLG* gene was associated with the ACM phenotype [74].

Overall, in our cohort, the prevalence of (L)P variants in uncommon genes was about 8% (3% in the first-level and 15% in the enlarged panels). This result supports the need for extended genetic testing including uncommon genes for such heterogeneous diseases as cardiomyopathies and channelopathies. Indeed, by limiting the genetic test to just phenotype-related (main) genes, we would have failed in identifying 13 mutations and the corresponding carrying patients (8% of the screened population). On the other hand, our approach was able to increase the diagnostic sensitivity of the test identifying these additional patients. 

Biotechnological research is always looking for new diagnostic strategies [75]. As NGS-based applications are currently widely used in diagnostic laboratories, and their costs are decreasing progressively, their use is desirable to enlarge the spectrum of the genes related to a specific disease. Indeed, accumulating data from different studies may allow for reconsidering the genotype–phenotype associations for some genes now considered to be uncommon.

In addition to pathogenic and likely pathogenic mutations, as expected in this kind of extended molecular screening, we identified many VUSs (41/82; 50% of the total number of identified variants) according to ACMG classification. Of the VUSs, 85% (35/41) were detected in uncommon genes. We detected variant c.764G>C in an HCM patient in the *FHL1* gene, which is classifiable as a VUS according to ACMG criteria. However, it is reported as pathogenic by HGMD database, showing strong evidence for a primary pathogenic role in HCM [73,76]. The *FHL1* gene was classified without evidence for a HCM association by ClinGen. The ACMG rule for classification is hypothesized to minimize the risk for false positive interpretations; however, it is also well-accepted that these rigorous criteria result in undercalling pathogenic variants in well-established cardiomyopathy genes. 

Certainly, VUS interpretation and the consequent clinical management of their carriers is a key challenge following NGS-based molecular testing [77]. The possibility to study a specific VUS in the context of a large pedigree by allowing for the robust analysis of the variant’s segregation with the clinical phenotype may be decisive to establish its pathogenic role; however, this is often unfeasible [78]. To definitively assess the molecular consequence of an identified VUS and thus establish its functional effects, the most proper approach should include in vitro functional assays [79,80,81,82]. Nevertheless, these functional evaluations are often difficult to be realized, especially in a routine setting and on a large scale, since different cellular models and different assays need to be used on the basis of the gene involved and on the type of molecular defect. The more that we enlarge the analyzed genomic region, the more the number of VUSs per patient increases. Nevertheless, even if VUS communication may be challenging and frustrating for both patients and clinicians, it offers a great opportunity for the re-evaluation of cases over time. Indeed, monitoring these variants over time may allow for their reclassification, and hence provide novel clues for the management of carriers and their relatives. Moreover, some of the VUSs may act as modifier genes, influencing a patient’s clinical phenotype, and may contribute to also explaining the observed clinical heterogeneity within families.

## 5. Conclusions

Considering all the above, extensive genetic test approaches are required to unravel the molecular bases of such a complex group of diseases as cardiomyopathies and channelopathies. NGS-based approaches, by allowing for the simultaneous analysis of multiple genes, increase the detection rate of causal mutations in inherited structural and electric hereditary cardiac diseases, and provide epidemiological data regarding the prevalence of causative mutations in uncommon genes. In addition, several uncertain or unknown variants (VUSs) are being identified and require more efforts in the near future to assess their pathogenicity.

The use of an NGS-based approach in diagnostics also increases the yield of mutations carriers’ identification in the rarest and uncommon genes, as shown in our study. Indeed, our data are a parameter of the increased diagnostic yield of such extended molecular analyses that also enable screening for rare and uncommon genes in a simple, reliable, and cost-saving manner.

## Figures and Tables

**Table 1:** List of genetic variants (n = 82) identified in the 133 patients of the study cohort.

Gene	Transcript	HGVS * Coding (cDNA)	HGVS * Protein Level	Patient’s Phenotype	ClinGen **	Variant Classification					
						Reference SNP ID	ClinVar	HGMD ^§^	ACMG ***	ACMG Supporting Criteria ^#^	gnomAD Frequency
*ABCC9*	NM_020297.4	c.2937G>A	p.Trp979Ter	HCM	NR	NR	NR	NR	P	PVS1/PM2/PP3	NF
*ANK2*	NM_001354273.1	c.187-3C>G	-	HCM	NR	rs1562805147	NR	NR	VUS	PM2/PP3	0.00000402
*ANK2*	NM_001354273.1	c.190G>A	p.Gly64Arg	BrS	DISPUTED	NR	NR	NR	VUS	PM2/PP3	NF
*CACNA1D*	NM_000720.4	c.4187C>A	p.Ala1396Asp	BrS	DISPUTED	rs745689505	NR	NR	VUS	PM2/PP3	NF
*CACNA1D*	NM_000720.4	c.1059C>G	p.Ala353Ala	HCM	NR	NR	NR	NR	VUS	PM2/BP7	NF
*CACNA2D1*	NM_001366867.1	c.1516-4C>A	-	BrS	DISPUTED	rs1371737796	NR	NR	VUS	PM2/BP4	NF
*CACNB2*	NM_201570.3	c.645G>T	p.Met215Ile	ACM	NR	NR	NR	NR	VUS	PM27PP3	NF
*CACNB2*	NM_201570.3	c.1652G>T	p.Arg551Met	ACM	NR	NR	NR	NR	VUS	PM2	NF
*CASQ2*	NM_001232.4	c.562C>T	p.His188Tyr	BrS	NR	NR	NR	P	VUS	PM2	NF
*CASQ2*	NM_001232.4	c.928G>A	p.Asp310Asn	BrS	NR	rs141314684	CI	P	LB	BS1/BP6	0.0007 vs. 0.00001 °
*CAV3*	NM_033337.3	c.233C>T	p.Thr78Met	HCM	NR	rs72546668	CI	P	B	PP3/PP2/BS1/BS2	0.00263 vs. 0.0004 °
*CAV3*	NM_033337.3	c.* 682delG	-	BrS	NR	NR	NR	NR	VUS	PM2/BP7	NF
*CTNNA3*	NM_013266.4	c.334C>T	p.Pro112Ser	HCM	NR	rs1485074194	NR	NR	VUS	PM2	NF
*DSC2*	NM_024422.6	c.926C>T	p.Ser 309Phe	BrS	NR	NR	NR	NR	VUS	PM2	NF
*DSP*	NM_004415.4	c.2848delA	p.Ile950LeufsTer27	ACM	DEFINITIVE	NR	P	P	P	PVS1/PM2	NF
*DSP*	NM_004415.4	c.5428C>T	p.Glu1810Ter	ACM	DEFINITIVE	rs397516946	LP	P	P	PM2/PVS1	NF
*DSP* ^§§^	NM_004415.4	c.8471_8483delGGTCCCGCTCCGG	p.Gly2824AlafsTer55	ACM	DEFINITIVE	NR	VUS	NR	P	PVS1/PM2	NF
*DSP*	NM_004415.4	c.8171A>G	p.Glu2724Arg	HCM	NR	NR	VUS	NR	VUS	PM2	NF
*FHL1*	NM_001159699.2	c.764G>C	p.Cys255Ser	HCM	NR	rs869025431	NR	P	LP	PM2/PP3/PP2/PP5	0.00000904
*FLNC*	NM_001127487.2	c.2830G>A	p.Val944Met	DCM	DEFINITIVE	NR	NR	NR	VUS	PM2/PP3	NF
*HCN4*	NM_005477.3	c.1748A>G	p.Asn583Ser	ACM	NR	rs1204195890	VUS	NR	VUS	PM2	0.0000131
*KCNE3*	NM_005472.5	c.-41+1G>C	-	ACM	NR	NR	NR	NR	LP	PVS1/PM2	NF
*KCNE3*	NM_005472.5	c.157C>T	p.Arg53Cys	BrS	DISPUTED	rs371666083	NR	NR	VUS	PM2	0.0000199
*KCNH2*	NM_000238.4	c.453dupC	p.Thr152HisfsTer180	LQTS	DEFINITIVE	rs761863251	CI	P	P	PVS1/PM2/PP5	0.000028 vs. 0.000008 °
*KCNH2*	NM_000238.4	c.2785dupG	p.Glu929GlyfsTer11	LQTS	DEFINITIVE	rs794728458	CI	P	P	PVS1/PP5/PM2	NF
*KCNQ1*	NM_181798.1	c.-62C>G	-	DCM	NR	NR	NR	NR	VUS	PM2/BP7	NF
*KCNQ1*	NM_000218.3	c.569G>T	p.Arg190Leu	LQTS	DEFINITIVE	rs120074178	P/LP	P	P	PS3/PM1/PM2/PP3/PP5/BP1	0.00000401
*KCNQ1*	NM_000218.3	c.877C>T	p.Arg293Cys	HCM	NR	rs199472737	CI	P	LP	PM1/PM5/PM2/PP3/PP5	0.000036
*KCNQ1*	NM_000218.3	c.1265delA	p.Lys422SerfsTer10	LQTS	DEFINITIVE	rs397508083	P/LP	P	P	PVS1/PP5/PM2	0.000012 vs. 0.000008 °
*KRAS*	NM_001369786.1	c.533C>G	p.Pro178Arg	BrS	NR	NR	NR	NR	VUS	PM2/PP2	NF
*KRT17*	NM_000422.3	c. 960+5G>A	-	LQTS	NR	rs370554150	P	NR	LP	PM2/PP3/PP5	0.000008
*LAMA3*	NM_198129.4	c.9685T>C	p-Ser3229Pro	LQTS	NR	rs765495036	NR	NR	VUS	PM2	0.00000398
*LDB3*	NM_001171610.2	c.860G>A	p.Gly287Glu	ACM	DISPUTED	NR	NR	NR	VUS	PM2	NF
*LMNA*	NM_001282626.2	c.949G>A	p.Glu317Lys	DCM	DEFINITIVE	rs56816490	P/LP	P	LP	PM1/PM2/PP3/PP5	0.00000657
*MYBPC3*	NM_000256.3	c.906-1G>C	-	BrS	NR	rs587776700	P	P	P	PVS1/PP5/PS3/PM2	0.00000433
*MYBPC3*	NM_000256.3	c.1591G>C	p.Gly531Arg	HCM	DEFINITIVE	rs397515912	LP	P	LP	PM1/PM2/PP3/PP5/BS2	0.0000121
*MYBPC3* ^§§§^	NM_000256.3	c.1790G>A	p.Arg597Gln	HCM	DEFINITIVE	rs727503195	CI	P	LP	PP3/PM1/PP5/BS2	0.00003
*MYBPC3*	NM_000256.3	c.1928-2A>G	-	HCM	DEFINITIVE	rs397515937	P	P	P	PVS1/PM2/PP5	NF
*MYBPC3*	NM_000256.3	c.2955G>T	p.Lys958Asn	HCM	DEFINITIVE	NR	NR	NR	VUS	PM2	0.00000657
*MYBPC3*	NM_000256.3	c.3627+2T>A	-	HCM	DEFINITIVE	rs1299079662	NR	P	LP	PVS1/PM2	0.00000405
*MYBPC3*	NM_000256.3	c.3775C>T	p.Gln1259Ter	HCM	DEFINITIVE	rs730880605	P	P	P	PVS1/PM2/PP3/PP5	NF
*MYH6*	NM_002471.4	c.5629G>A	p.Val1877Ile	ACM	NR	NR	NR	NR	VUS	PM2	NF
*MYH7*	NM_000257.4	c.1615A>C	p.Met539Leu	HCM	DEFINITIVE	rs730880930	LP	P	LP	PM1/PM2/PP2/PP3/PP5/BP1	NF
*MYH7*	NM_000257.4	c.2155C>T	p. Arg719Trp	HCM	DEFINITIVE	rs121913637	P	P	LP	PM1/PM2/PP2/PP3/PP5/BP1	0.00000657
*MYH7*	NM_000257.4	c.4066G>A	p.Glu1356Lys	HCM	DEFINITIVE	rs727503246	LP	P	LP	PM1/PM2/PP2/PP3/PP5/BP1	0.00000657
*MYH7* ^#^	NM_000257.4	c.4130C>T	p.Thr1377Met	HCM	DEFINITIVE	rs397516201	LP	P	LP	PM1/PM2/PP2/PP3/PP5/BP1	0.00000398
*MYL2*	NM_000432.4	c.484G>A	p.Gly162Arg	HCM	DEFINITIVE	rs199474814	CI	P	P	PM1/PM2/PP2/PM5/PP3/PP5	NF
*OBSCN*	NM_001271223.2	c.22911_22912delGT	p.Ser7638MetfsTer30	HCM	NR	rs1558418533	NR	NR	LP	PVS1/PM2	0.00000402
*PKP2*	NM_004572.4	c.368G>A	p.Trp123Ter	ACM	DEFINITIVE	rs760576804	P	P	P	PVS1/PM2/PP3/PP5	NF
*PKP2*	NM_004572.4	c.948_949delAG	p.Arg316SerfsTer19	ACM	DEFINITIVE	NR	NR	NR	LP	PVS1/PM2	NF
*PKP2*	NM_004572.4	c.1378+1G>C	-	HCM	NR	rs397516994	P/LP	P	P	PVS1/PP5/PM2	0.00000399
*PLN#*	NM_002667.5	c.40_42delAGA	p.Arg14del	ACM/DCM	DEFINITIVE in DCM MODERATE in ACM	rs397516784	CI	P	LP	PS3/PM2/PM4/PP5	0.00000398
*POLG*	NM_002693.3	c.530G>A	p.Arg177Gln	LQTS	NR	NR	NR	NR	VUS	PM2/PP2	NF
*POLG*	NM_002693.3	c.752C>T	p.Thr251Ile	ACM	NR	rs113994094	CI	P	LP	PM2/PP2/PP5	0.00155 vs. 0.00009 °
*POLG*	NM_002693.3	c.1402A>G	p.Asn468Asp	BrS	NR	rs145843073	CI	P	LP	PP5/PM2/BP4	0.000473 vs. 0.00001 °
*POLG*	NM_002693.3	c.1760C>T	p.Pro587Leu	ACM	NR	rs113994096	CI	P	P	PS3/PM2/PP5/PP3	0.00155 vs. 0.00009 °
*PRDM16*	NM_022114.4	c.1400C>A	p.Pro467His	BrS	NR	NR	NR	NR	VUS	PM2	NF
*RAF1*	NM_001354689.3	c.709G>A	p.Ala237Thr	HCM	NR	rs587777588	P	P	LP	PM2/PM1/PP5	0.0000159
*RAF1*	NM_001354689.3	c.945T>G	p.Ser315Arg	HCM	NR	NR	NR	NR	VUS	PM2/PP2	NF
*RYR2*	NM_001035.3	c.5475C>T	p.Phe1825Phe	DCM	NR	NR	NR	NR	VUS	PM2/BP7	NF
*RYR2*	NM_001035.3	c.8215A>G	p.Asn2739Asp	HCM	LIMITED	NR	NR	NR	VUS	PM2/PP2	NF
*RYR2*	NM_001035.3	c.13457C>G	p.Ala4486Gly	ACM	REFUTED	rs779309213	VUS	NR	VUS	PM2/PP2	0.0000122
*SCN10A*	NM_006514.3	c.3088C>T	p.Gln1030Ter	HCM	NR	rs778772059	NR	NR	VUS	PM2	0.00000409
*SCN10A*	NM_006514.3	c.4948A>T	p.Ser1650Cys	HCM	NR	rs780649338	VUS	NR	VUS	PM2/PP3	0.0000159
*SCN3B*	NM_001040151.2	c.354C>T	p.Tyr118Tyr	LQTS	NR	NR	NR	NR	VUS	PM2/BP7	0.00000657
*SCN5A*	NM_198056.3	c.2441G>A	p.Arg814Gln	BrS	DEFINITIVE	rs199473584	CI	P	LP	PS3/PM5/PP3/PP5	0.0000242 vs. 0.00001 °
*SCN5A*	NM_198056.3	c.4414_4416delAAC	p.Asn1472del	LQTS	DEFINITIVE	NR	NR	P	LP	PM1/PM2/PM4	NF
*SCN5A*	NM_198056.3	c.5494C>G	p.Gln1832Glu	BrS	DEFINITIVE	rs199473320	CI	P	VUS	BP6	0.0000601 vs. 0.00001 °
*SGCD*	NM_000337.6	c.451T>G	p.Ser151Ala	BrS	NR	rs121909298	VUS	P	VUS	PP3	0.000202 vs. 0.00001 °
*TBX1*	NM_080647.1	c.684+7G>T	-	DCM	NR	NR	NR	NR	VUS	PM2/BBP4	NF
*TGFB3*	NM_001329939.1	c.8T>C	p.Met3Thr	BrS	NR	NR	NR	NR	VUS	PM2/PP3	NF
*TGFB3*	NM_001329939.1	c.* 495C>T	-	HCM	NR	rs387906514	P	P	VUS	PM2	0.00000667
*TNNI3*	NM_000363.5	c.434G>A	p.Arg145Gln	HCM	DEFINITIVE	rs397516349	P/LP	P	P	PP5/PS3/PM1/PM5/PM2/PP3	0.0000161
*TNNI3*	NM_000363.5	c.485G>A	p.Arg162Gln	HCM	DEFINITIVE	rs397516354	P/LP	P	P	PP5/PM1/PM5/PM2/PP3	0.0000402
*TNNI3K*	NM_015978.3	c.2187 G>T	p.Met729Ile	HCM	DEFINITIVE	rs1372831124	NR	NR	VUS	PM2	0.00000404
*TNNT2*	NM_001276347.2	c.388C>T	p.Arg130Cys	HCM	DEFINITIVE	rs397516463	NR	P	P	PM1/PM2/PP2/PP3/PP5	NF
*TRPM4*	NM_017636.4	c.3329-2A>G	-	DCM	NR	rs751095080	NR	NR	LP	PVS1/PM2	0.00000401
*TTN*	NM_001267550.2	c.8854_ 8874delACATTTGTCTGTGGCAATGAC	p.Thr2952_Asp2958del	DCM	DEFINITIVE	NR	NR	NR	VUS	PM2/PM4	NF
*TTN*	NM_001267550.2	c.50255C>G	p.Pro16752Arg	HCM	LIMITED	NR	NR	NR	VUS	PM2	NF
*TTN*	NM_001267550.2	c.83378A>G	p.Asp27793Gly	DCM	DEFINITIVE	NR	NR	NR	VUS	PM2	NF
*TTN*	NM_001267550.2	c.69500G>A	p.Gly23167Asp	HCM	LIMITED	rs747019187	NR	NR	VUS	PM2	NF
*TTN*	NM_001267550.2	c.102565G>C	p.Asp34189His	BrS	NR	NR	NR	NR	VUS	PM2	NF

For each genetic variant, the following information is shown: reference transcript, variant nomenclature at DNA and protein level according to Human Genome Variation Society (* HGVS) guidelines, clinical phenotype, gene classification according to the NIH-funded Clinical Genome Resource (** ClinGen) with respect to the clinical phenotype, reference singe-nucleotide polymorphism (SNP) ID number (rs), clinical significance by the ClinVar database, HGMD database (^§^ Human Genetic Mutation Database (HGMD) and American College of Medical Genetics (*** ACMG) classification, including ACMG supporting criteria (**^#^**) according to [62]; variant allele frequency according to gnomAD exome database. ^§§^ Mutations found in 2 patients; ^§§§^ mutations found in 3 patients; ^#^ mutations found in 2 patients; ° GnomAD frequency versus the maximal tolerated allele frequency for the specific cardiomyopathy. NF: not found; NR: not reported; LP: likely pathogenic; P: pathogenic; LB: likely benign; VUS: variant of uncertain significance; CI: conflicting interpretation; ACM: arrhythmogenic cardiomyopathy; DCM: dilated cardiomyopathy; HCM: hypertrophic cardiomyopathy; BrS: Brugada syndrome; LQTS: long QT syndrome.

**Table 2:** Demographic, clinical, and instrumental data of patients carrying pathogenic or likely pathogenic variants in uncommon genes with respect to the clinical diagnosis or suspicion of an inherited cardiac disease (cardiomyopathy or channelopathy).

ID	Age at Diagnosis (Years)	Sex	Family History	Symptoms *	ECG *	Gene	Nucleotide Variant (Aminoacid Change)	Echocardiography/CMR **	Definitive Diagnosis
NA01	20	M	Negative for SCD and HCM	Exertional dyspnoea (NYHA Class II)	Signs of LVH, diffuse repolarization abnormalities	*ABCC9*	c.2937G>A (p.Trp979Ter)	Symmetric LVH with MWT (18 mm) at the level of the basal IVS and first-degree diastolic dysfunction	HCM
NA02	47	M	Negative for SCD, and ACM	Asymptomatic	Diffuse repolarization abnormalities	*KCNE3*	c.-41+1G>C	Regional dyskinesia of right ventricle free wall. Identification of regional fibrosis with LGE	ACM
NA03	44	M	Negative for SCD and HCM	Asymptomatic	Right axis deviation; delayed intraventricular left conduction	*KCNQ1*	c.877C>T (p.Arg293Cys)	Symmetric LVH with MWT (22 mm) at the level of the basal IVS	HCM
NA04	51	F	Negative for SCD and BrS	Syncope	Brugada type 1 pattern	*MYBPC3*	c.906-1G>C	Normal wall thicknesses and chamber diameters	BrS
NA05	79	M	Negative for SCD and HCM	Asymptomatic	diffuse repolarization abnormalities	*PKP2*	c.1378+1G>C	Asymmetrical LVHMWT: 19 mm	HCM
NA06	9	M	Negative for SCD and BrS	Asymptomatic	Flecainide-induced Brugada type 1 pattern	*POLG*	c.1402A>G (p.Asn468Asp)	Normal wall thicknesses and chamber diameters	BrS
NA07	20	M	Positive for SCD and ACM	Syncope	Diffuse repolarization abnormalities	*POLG*	c.752C>T (p.Thr251Ile); c.1760C>T (p.Pro587Leu)	Normal wall thicknesses and chamber diameters. Presence of diffuse late gadolinium enhancement with subepicardial distribution in the basal segment of the inferior and lateral walls at CMR	ACM
NA08	18	M	Negative for SCD and HCM	Exertional dyspnoea (NYHA Class II)	Signs of LVH, negative T-waves in aVL, V5, V6 leads	*RAF1*	c.709G>A (p.Ala237Thr)	Asymmetric obstructive LVH with MWT (15 mm) at the level of the basal IVS	HCM

Abbreviations: ACM, arrhythmogenic cardiomyopathy; BrS, Brugada syndrome; HCM, hypertrophic cardiomyopathy; LVH, left ventricular hypertrophy; LQTS, long QT syndrome; ECG, electrocardiogram; CMR, cardiac magnetic resonance; IVS, interventricular septum; MWT, maximal wall thickness; NYHA, New York Heart Association; SCD, sudden cardiac death; LGE, late gadolinium enhancement. * At first evaluation; ** at last follow-up.

## Data Availability

Data are reported within the text.

## Supplementary Materials

The following supporting information can be downloaded at: https://www.mdpi.com/article/10.3390/biom12101417/s1. Table S1: genes reported in the literature associated with cardiomyopathies and channelopathies.

## Abbreviations

NGS	Next-generation sequencing
VUS	Variants of unknown significance
SCD	Sudden cardiac death
HCM	Hypertrophic cardiomyopathy
ACM	Arrhythmogenic cardiomyopathy
DCM	Dilated cardiomyopathy
LQTS	Long QT syndrome
BrS	Brugada syndrome
SQTS	Short QT syndrome
CPVT	Catecholaminergic polymorphic ventricular tachycardia
*MYBPC3*	Cardiac myosin binding protein 3
*MYH7*	β-myosin heavy chain
*TNNT2*	Cardiac troponin T
*TNNI3*	Cardiac troponin I
*TPM1*	α-tropomyosin
*ACTC1*	Cardiac α-actin
*MYL2*	Myosin regulatory light chain
MYL3	Myosin essential light chain
*MYOZ2*	Myozenin 2 gene
*ACTN2*	α-actinin-2
*TTN*	Titin
*MYOM1*	Myomesin 1
*ANKRD1*	Cardiac ankyrin repeat protein 1
*TCAP*	Telethonin
*PKP2*	Plakophilin
*DSP*	Desmoplakin
*DSC2*	Desmocollin 2
*DSG2*	Desmoglein
*TMEM43*	Transmembrane protein 43
*PLN*	Phospholamban
*CDH2*	Cadherin-2
*CTNNA3*	Catenin alpha 3
*FLNC*	Filamin C
*TJP1*	Tight junction protein 1
*ANK2*	Ankyrin 2
*TP63*	Tumor protein P63
*SCN10A*	sodium voltage-gated channel alpha subunit 10
*CACNA1C*	Calcium voltage-gated channel subunit alpha1 c
*ABCC9*	ATP binding cassette subfamily c member 9
*SCN1B*	Sodium voltage-gated channel beta subunit 1
*KCNH2*	Potassium voltage-gated channel subfamily h member 2
*CACNB2*	Calcium voltage-gated channel auxiliary subunit beta 2
*TRPM4*	Transient receptor potential cation channel subfamily m member 4
*ANK3*	Ankyrin 3
*CACNA2D1*	Calcium voltage-gated channel auxiliary subunit alpha2delta 1
*FGF12*	Fibroblast growth factor 12
*GPD1L*	Glycerol-3-phosphate dehydrogenase 1 like
*HCN4*	Hyperpolarization activated cyclic nucleotide gated potassium channel 4
*KCNQ1*	Potassium voltage-gated channel subfamily q member 1
*KCNH2*	Potassium voltage-gated channel subfamily h member 2
*SCN5A*	Sodium voltage-gated channel alpha subunit 5
*AKAP9*	a-Kinase anchoring protein 9
*RYR2*	Ryanodine receptor 2
*CASQ2*	Calsequestrin 2
*CALM1*	Calmodulin 1
*CALM2*	Calmodulin 2
*CALM3*	Calmodulin 3
*KCNJ2*	Potassium inwardly rectifying channel subfamily j member 2
*TRDN*	Triadin
ACMG	American College of Medical Genetics and Genomics
*RAF1*	RAF-1 proto-oncogene, serine/threonine kinase
*LDLR*	Low-density lipoprotein receptor
*GAA*	Glucosidase alpha, acid
LP	Likely pathogenic
*FHL1*	Four and a half LIM domains 1
P	Pathogenic
LP	Likely pathogenic
B	Benign
LB	Likely benign
ESC	European Society of Cardiology
AHA	American Heart Association
ACC	American College of Cardiology
