# Supplementary material for: Next-Generation Sequencing Gene Panels in Inheritable Cardiomyopathies and Channelopathies: Prevalence of Pathogenic Variants and Variants of Unknown Significance in Uncommon Genes
Source: Biomolecules. 2022 Oct 3;12(10):1417. doi: 10.3390/biom12101417 (PMC9599286; doi:10.3390/biom12101417)
Supplement: Supplementary file 1 [file biomolecules-12-01417-s001.zip › biomolecules-1861539-supplementary.pdf]

Supplemental Table S1. Genes reported in literature as associated with cardiomyopathies and channelopathies

DISEASE	Gene OMIM id	GENE	CYTOGENETIC BAND	PROTEIN	CLIN GENE	INHERITANCE
Brugada Syndrome (BrS)	601439	*ABCC9*	12p12.1	ATP-Binding Cassette, Subfamily C, Member 9	N.R.	AD
	600465	*ANK3*	10q21.2	Ankyrin 3 (G)	N.R.	AD
	611875	*CACNA1C*	12p13.33	α subunit α1C of the Cav1.2 calcium channel	Disputed	AD
	114204	*CACNA2D1*	7q21.11	δ subunit Cavα2δ1 of calcium channel	Disputed	AD
	611876	*CACNB2*	10p12.33-p12.31	β subunit Cavβ2b of calcium channel	Disputed	AD
	601513	*FGF12*	3q28-q29	Fibroblast growth factor 12	N.R.	AD
	611777	*GPD1L*	3p22.3	Glycerol-3-phosphate dehydrogenase 1-like	Disputed	AD
	613123	*HCN4*	15q24.1	Hyperpolarization-activated cyclic nucleotide-gated channel 4	Disputed	AD
	604674	*HEY2*	6q22	Hairy/Enhancer of Split-related with YRPW motif 2	N.R.	AD
	605410	*KCND2*	7q31.31	α subunit of the KV4.2 potassium channel	N.R.	AD
	616399	*KCND3*	1p13.2	α subunit of the KV4.3 potassium channel	Disputed	AD
	613119	*KCNE3*	11q13.4	β subunit MiRP2 of potassium channel	Disputed	AD
	152427	*KCNH2*	7q36.1	α subunit of the HERG potassium channel	Disputed	AD
	600935	*KCNJ8*	12p12.1	α subunit of the KIR6.1 potassium channel	Disputed	AD
	602861	*PKP2*	12p11.21	Plakophilin 2	Disputed	AD
	607954	*RANGRF*	17p13.1	RAN guanine nucleotide release factor	Disputed	AD
	604427	*SCN10A*	3p22.2	α subunit of the Nav1.8 sodium channel	Disputed	AD
	612838	*SCN1B*	19q13.12	β subunit Navβ1 of sodium channel	Disputed	AD
	601327	*SCN2B*	11q23.3	β subunit Navβ2 of sodium channel	Disputed	AD
	613120	*SCN3B*	11q24.1	β subunit Navβ3 of sodium channel	Disputed	AD
	601144	*SCN5A*	3p22.2	α subunit of the Nav1.5 sodium channel	Definitive	AD
	603961	*SEMA3A*	7q21.11	Semaphorin family protein	N.R.	AD
	602701	*SLMAP*	3p14.3	Sarcolemma-associated protein	Disputed	AD
	606936	*TRPM4*	19q13.33	Calcium-activated non-selective ion channel	Disputed	AD
Long QT Syndrome (LQT)	604001	*AKAP9*	7q21.2	A-kinase anchor protein 9	Disputed	AD
	106410	*ANK2*	4q25-q26	Ankyrin 2 (B)	N.R.	AD
	114205	*CACNA1C*	12p13.33	α subunit α1C of the Cav1.2 calcium channel	Moderate	AD
	114180	*CALM1*	14q32.11	Calmodulin 1	Definitive	AD
	114182	*CALM2*	2p21	Calmodulin 2	Definitive	AD
	114183	*CALM3*	19q13.32	Calmodulin 3	Definitive	AD
	601253	*CAV3*	3p25.3	Caveolin 3	N.R.	AD
	176261	*KCNE1*	21q22.11-q22.12	Potassium voltage-gated channel subfamily E regulatory subunit 1	Limited	AD/AR
	603796	*KCNE2*	21q22.11	Potassium voltage-gated channel subfamily E regulatory subunit 2	Disputed	AD
	152427	*KCNH2*	7q36.1	α subunit of the HERG potassium channel	Definitive	AD
	600681	*KCNJ2*	17q24.3	Potassium inwardly-rectifying channel, subfamily J, member 2	Limited	AD/AR
	600734	*KCNJ5*	11q24.3	Potassium inwardly-rectifying channel, subfamily J, member 5	Disputed	AD
	607542	*KCNQ1*	11p15.5-p15.4	Kv7.1 hERG/Kv11.1	Definitive	AD/AR
	602235	*KCNQ2*	20q13.33	Potassium voltage-gated channel subfamily Q member 2	N.R.	AD
	180902	*RYR2*	1q43	Ryanodine Receptor 2	N.R.	AD
	600235	*SCN1B*	19q13.12	Sodium voltage-gated channel beta subunit 1	N.R.	AD
	608256	*SCN4B*	11q23.3	Sodium voltage-gated channel beta subunit 4	Disputed	AD
	600163	*SCN5A*	3p22.2	α subunit of the Nav1.5 sodium channel	Definitive	AD
	601017	*SNTA1*	20q11.21	α1-Syntrophin	Disputed	AD
	603283	*TRDN*	6q22.31	Triadin	Strong	AR
Short QT Syndrome (SQT)	611875	*CACNA1C*	12p13.33	α subunit α1C of the Cav1.2 calcium channel	Disputed	AD
	114204	*CACNA2D1*	7q21.11	δ subunit Cavα2δ1 of calcium channel	Disputed	AD
	611876	*CACNB2*	10p12.33-p12.31	β subunit Cavβ2b of calcium channel	Disputed	AD
	152427	*KCNH2*	7q36.1	α subunit of the HERG potassium channel	Definitive	AD
	600681	*KCNJ2*	17q24.3	Potassium inwardly-rectifying channel, subfamily J, member 2	Moderate	AD/AR
	607542	*KCNQ1*	11p15.5-p15.4	Potassium voltage gated channel, KQT-like subfamily, member 1	Strong	AD
	601144	*SCN5A*	3p22.2	α subunit of the Nav1.5 sodium channel	Disputed	AD
	106195	*SLC4A3*	2q35	Solute carrier family 4 (anion exchanger), member 3	Moderate	AD
Catecholaminergic Polymorphic Ventricular Tachycardia (CPVT)	106410	*ANK2*	4q25-q26	Ankyrin 2 (B)	Disputed	AD
	114180	*CALM1*	14q32.11	Calmodulin 1	Moderate	AD
	114182	*CALM2*	2p21	Calmodulin 2	Moderate	AD
	114183	*CALM3*	19q13.32	Calmodulin 3	Moderate	AD
	114251	*CASQ2*	1p13.1	Calsequestrin 2	Definitive	AR
	600681	*KCNJ2*	17q24.3	Potassium inwardly-rectifying channel, subfamily J, member 2	Disputed	AR
	180902	*RYR2*	1q43	Ryanodine Receptor 2	Definitive	AD
	603283	*TRDN*	6q22.31	Triadin	Definitive	AR
omyopathy (ACM)	106410	*ANK2*	4q25-q26	Ankyrin 2 (B)	N.R.	AD
	114020	*CDH2*	18q12.1	Cadherin 2 - N-Cadherin	Limited	AD
	607667	*CTNNA3*	10q22.2	Catenin, alpha 3	Limited	AD
	125660	*DES*	2q35	Desmin	Moderate	AD
	125645	*DSC2*	18q12.1	Desmocollin 2	Definitive	AD/AR
	125671	*DSG2*	18q12.1	Desmoglein 2	Definitive	AD
	125647	*DSP*	6p24	Desmoplakin	Definitive	AD
	102565	*FLNC*	7q32.1	Filamin C	N.R.	AD
	173325	*JUP*	17q21	Junction plakoglobin	Definitive	AD

Arrhythmogenic Cardi	150330	*LMNA*	1q22	Lamin A/C	Limited	AD
	602861	*PKP2*	12p11.21	Plakophilin 2	Definitive	AD
	172405	*PLN*	6q22.31	Phospholamban	Moderate	AR
	180902	*RYR2*	1q43	Ryanodine receptor 2	Refuted	AD
	601144	*SCN5A*	3p22.2	α subunit of the Nav1.5 sodium channel	Limited	AD
	190230	*TGFB3*	14q24.3	Transforming Growth Factor β 3	Limited	AD
	612658	*TJP1*	15q13.1	Tight Junction Protein	Limited	AD
	612048	*TMEM43*	3p25.1	Transmembrane protein 43	Definitive	AD
	603273	*TP63*	3q28	Tumor Protein p63	N.R.	AD
	188840	*TTN*	2q31.2	Titin	Limited	AD
Dilated Cardiomyopathy (DCM)	601439	*ABCC9*	12p12.1	ATP-Binding Cassette, Subfamily C, Member 9	Limited	AD
	602330	*ABLIM1*	10q25.3	Limatin (actin-binding LIM domain protein)	N.R.	AD
	102540	*ACTC1*	15q14	Actin, alpha, cardiac muscle	Moderate	AD
	102573	*ACTN2*	1q43	Alpha-actinin 2	N.R.	AD
	606844	*ALMS1*	2p13.1	ALMS1 centrosome and basal body associated protein	N.R.	AR
	609599	*ANKRD1*	10q23.31	Ankyrin repeat domain-containing protein 1	Limited	AD
	608662	*ANO5*	11p14.3	Anoctamin 5	N.R.	AR
	603883	*BAG3*	10q26.1	BCL2-associated athanogene	Definitive	AD
	611414	*CALR3*	19p13.11	Calreticulin 3	N.R.	AD
	114251	*CASQ2*	1p13.1	Calsequestrin 2	N.R.	AD
	601253	*CAV3*	3p25.3	Caveolin 3	N.R.	AD
	123590	*CRYAB*	11q23.1	Alpha B crystallin	N.R.	AD
	600824	*CSRP3*	11p15.1	Cysteine- and glycine-rich protein 3	Limited	AD
	600435	*CTF1*	16p11.2	Cardiotrophin 1	Limited	AD
	128239	*DAG1*	3p21.31	Dystroglycan, alpha	N.R.	AR
	125660	*DES*	2q35	Desmin	Definitive	AD
	300377	*DMD*	Xp21.2-p21.1	Dystrophin	N.R.	XLR
	605377	*DMPK*	19q13.32	Dystrophia myotonica protein kinase gene	N.R.	AD
	610746	*DOLK*	9q34.11	Dolichol Kinase	N.R.	AR
	125645	*DSC2*	18q12.1	Desmocollin 2	N.R.	AD
	125671	*DSG2*	18q12.1	Desmoglein 2	Limited	AD
	125647	*DSP*	6p24	Desmoplakin	Strong	AD
	601239	*DTNA*	18q12.1	Dystrobrevin, alpha	Limited	AD/AR
	300384	*EMD*	Xq28	Emerin	N.R.	XLR
	603550	*EYA4*	6q23.2	Eyes absent 4	Limited	AD
	300163	*FHL1*	Xq26.3	Four-and –a-half LIM domains 1	N.R.	XLD/XLR
	602633	*FHL2*	2q12.2	Four-and –a-half LIM domains 2	N.R.	AD
	102565	*FLNC*	7q32.1	Filamin C	Definitive	AD
	614518	*GATAD1*	7q21.2	GATA Zinc Finger Domain Containing Protein 1	Limited	AD
	300644	*GLA*	Xq22.1	Galactosidase, alpha	N.R.	XLR
	602366	*ILK*	11p15.4	Integrin-linked kinase	Limited	AD
	173325	*JUP*	17q21	Junction plakoglobin	N.R.	AD
	156225	*LAMA2*	6q22.33	Laminin Alpha, 2	N.R.	AR
	600133	*LAMA4*	6q21	Laminin Alpha, 4	Limited	AD
	309060	*LAMP2*	Xq24	Lysosome-associated membrane protein 2	N.R.	XLR
	605906	*LDB3*	10q23.2	LIM domain-binding 3	Limited	AD
	150330	*LMNA*	1q22	Lamin A/C	Definitive	AD
	600958	*MYBPC3*	11p11.2	Myosin-binding protein C, cardiac	Limited	AD
	160710	*MYH6*	14q11.2	Alpha-myosin heavy chain 6	Limited	AD
	160760	*MYH7*	14q11.2	Myosin, heavy chain 7, cardiac muscle, beta	Definitive	AD
	160781	*MYL2*	12q24.11	Myosin light chain 2	Limited	AD
	160790	*MYL3*	3p21.31	Myosin light chain 3	Disputed	AD/AR
	603508	*MYOM1*	18p11.31	Myomesin 1	N.R.	Unknown
	605603	*MYOZ1*	10q22.2	Myozenin 1	N.R.	AD/AR
	605602	*MYOZ2*	4q26	Myozenin 2	N.R.	AD
	608517	*MYPN*	10q21.3	Myopalladin	Limited	AD
	605491	*NEBL*	10p12.31	Nebulette	Limited	AD
	613121	*NEXN*	1p31.1	Nexilin	Moderate	AD
	600584	*NKX2-5*	5q35.1	NK2 homeobox 5; cardiac specific homeobox 1	Limited	AD
	605900	*PDLIM1*	10q23.33	C-terminal LIM domain protein 1	N.R.	AD
	605889	*PDLIM3*	4q35.1	PDZ and LIM domain protein 3	Disputed	AD
	603422	*PDLIM4*	5q31.1	PDZ and LIM domain protein 4	N.R.	AD
	602861	*PKP2*	12p11.21	Plakophilin 2	Disputed	AD
	172405	*PLN*	6q22.31	Phospholamban	N.R.	AR
	605557	*PRDM16*	1p36.32	PR domain containing 16	Limited	AD
	602743	*PRKAG2*	7q36.1	Protein Kinase, AMP-Activated, Non-Catalytic, Gamma 2	N.R.	AD
	104311	*PSEN1*	14q24.2	Presenilin 1	Disputed	AD
	600759	*PSEN2*	1q42.13	Presenilin 2	Limited	AD
	176876	*PTPN11*	12q24.13	Protein-Tyrosine Phosphatase, Non-Receptor Type, 11	N.R.	AD
	613171	*RBM20*	10q25.2	RNA-binding motif protein 20	Definitive	AD
	609591	*RIT1*	1q22	RIC-like protein without CAAX motif 1	N.R.	AD
	180902	*RYR2*	1q43	Ryanodine Receptor 2	N.R.	AD
	601144	*SCN5A*	3p22.2	α subunit of the Nav1.5 sodium channel	Definitive	AD
	601411	*SGCD*	5q33.2-q33.3	Delta-sarcoglycan	Limited	AD
	603377	*SLC22A5*	5q31.1	Solute Carrier Family 22 (Organic Cation Transporter), Member 5	N.R.	AR

	601017	*SNTA1*	20q11.21	α1-Syntrophin	N.R.	AD
	182530	*SOS1*	2p22.1	SOS Ras/Rac guanine nucleotide exchange factor 1	N.R.	AD
	607723	*SUN1*	7p22.3	SAD1 and UNC84 domain-containing protein 1	N.R.	AD
	613569	*SUN2*	22q13.1	SAD1 and UNC84 domain-containing protein 2	N.R.	AD
	608441	*SYNE1*	6q25.2	Nesprin 1, Synaptic nuclear envelop protein 1	N.R.	AD
	608442	*SYNE2*	14q23.2	Nesprin 2, Synaptic nuclear envelop protein 2	N.R.	AD
	300394	*TAZ*	Xq28	Tafazzin	N.R.	XLR
	601620	*TBX5*	12q24.21	T-box 5	N.R.	AD
	604488	*TCAP*	17q12	Titin-cap; telethonin	Limited	AD/AR
	603306	*TCF21*	6q23.2	Transcription factor 21, epicardin	N.R.	AD
	190230	*TGFB3*	14q24.3	Transforming Growth Factor β 3	N.R.	AD
	612048	*TMEM43*	3p25.1	Transmembrane protein 43	N.R.	AD
	188380	*TMPO*	12q23.1	Thymopoietin	N.R.	AD
	191040	*TNNC1*	3p21.1	Cardiac troponin C	Definitive	AD
	191044	*TNNI3*	19q13.42	Cardiac troponin I3	Moderate	AD
	191045	*TNNT2*	1q32.1	Cardiac troponin T2	Definitive	AD
	191010	*TPM1*	15q22.2	Tropomyosin 1	Moderate	AD
	188840	*TTN*	2q31.2	Titin	Definitive	AD
	176300	*TTR*	18q12.1	Transthyretin	N.R.	AD
	193065	*VCL*	10q22.2	Vinculin	Moderate	AD
Hypertrophic Cardiomyopathy (HCM)	102540	*ACTC1*	15q14	Actin, alpha, cardiac muscle	Definitive	AD
	102573	*ACTN2*	1q43	Alpha-actinin 2	Moderate	AD
	617608	*ALPK3*	15q25.3	Alpha kinase 3	Strong	AR
	603883	*BAG3*	10q26.1	BCL2-associated athanogene	N.R.	AD
	611414	*CALR3*	19p13.11	Calreticulin 3	Limited	AD
	601253	*CAV3*	3p25.3	Caveolin 3	N.R.	AD
	123590	*CRYAB*	11q23.1	Alpha B crystallin	N.R.	AD
	600824	*CSRP3*	11p15.1	Cysteine- and glycine-rich protein 3	Moderate	AD
	125660	*DES*	2q35	Desmin	N.R.	AD
	300163	*FHL1*	Xq26.3	Four-and –a-half LIM domains 1	N.R.	XLRD/XLRR
	602633	*FHL2*	2q12.2	Four-and –a-half LIM domains 2	N.R.	AD
	102565	*FLNC*	7q32.1	Filamin C	Definitive	AD
	300644	*GLA*	Xq22.1	Galactosidase, alpha	N.R.	XLR
	602366	*ILK*	11p15.4	Integrin-linked kinase	N.R.	AD
	605267	*JPH2*	20q13.12	Junctophilin 2	Moderate	AD
	309060	*LAMP2*	Xq24	Lysosome-associated membrane protein 2	N.R.	XLR
	605906	*LDB3*	10q23.2	LIM domain-binding 3	N.R.	AD
	600958	*MYBPC3*	11p11.2	Myosin-binding protein C, cardiac	Definitive	AD
	160710	*MYH6*	14q11.2	Alpha-myosin heavy chain 6	Limited	AD
	160760	*MYH7*	14q11.2	Myosin, heavy chain 7, cardiac muscle, beta	Definitive	AD
	609928	*MYH7B*	20q11.22	Myosin Heavy Chain 7B	N.R.	AD
	160781	*MYL2*	12q24.11	Myosin light chain 2	Definitive	AD
	160790	*MYL3*	3p21.31	Myosin light chain 3	Definitive	AD/AR
	606566	*MYLK2*	20q11.21	Myosin light chain kinase 2	Limited	AD
	605603	*MYOZ1*	10q22.2	Myozenin 1	N.R.	AD/AR
	605602	*MYOZ2*	4q26	Myozenin 2	Limited	AD
	608517	*MYPN*	10q21.3	Myopalladin	Limited	AD
	613121	*NEXN*	1p31.1	Nexilin	Limited	AD
	605900	*PDLIM1*	10q23.33	C-terminal LIM domain protein 1	N.R.	AD
	605889	*PDLIM3*	4q35.1	PDZ and LIM domain protein 3	Limited	AD
	603422	*PDLIM4*	5q31.1	PDZ and LIM domain protein 4	N.R.	AD
	172405	*PLN*	6q22.31	Phospholamban	Definitive	AD
	602743	*PRKAG2*	7q36.1	Protein Kinase, AMP-Activated, Non-Catalytic, Gamma 2	Definitive	AD
	176876	*PTPN11*	12q24.13	Protein-Tyrosine Phosphatase, Non-Receptor Type, 11	N.R.	AD
	604488	*TCAP*	17q12	Titin-cap; telethonin	Limited	AD /AR
	612418	*TMEM70*	8q21.11	Mitochondrial complex V (ATP synthase) deficiency, nuclear type 2	N.R.	AR
	191040	*TNNC1*	3p21.1	Cardiac troponin C	Moderate	AD
	191044	*TNNI3*	19q13.42	Cardiac troponin I3	Definitive	AD
	191045	*TNNT2*	1q32.1	Cardiac troponin T2	Definitive	AD
	191010	*TPM1*	15q22.2	Tropomyosin 1	Definitive	AD
	188840	*TTN*	2q31.2	Titin	Limited	AD
	176300	*TTR*	18q12.1	Transthyretin	N.R.	AD
	193065	*VCL*	10q22.2	Vinculin	limited	AD
Restrictive Cardiomyopathy(RCM)	102540	*ACTC1*	15q14	Actin, alpha, cardiac muscle	N.R.	AD
	123590	*CRYAB*	11q23.1	Alpha B crystallin	N.R.	AD
	125660	*DES*	2q35	Desmin	N.R.	AD
	160760	*MYH7*	14q11.2	Myosin, heavy chain 7, cardiac muscle, beta	N.R.	AD
	160790	*MYL3*	3p21.31	Myosin light chain 3	N.R.	AD/AR
	608517	*MYPN*	10q21.3	Myopalladin	N.R.	AD
	191040	*TNNC1*	3p21.1	Cardiac troponin C	N.R.	AD
	191044	*TNNI3*	19q13.42	Cardiac troponin I3	N.R.	AD
	191045	*TNNT2*	1q32.1	Cardiac troponin T2	N.R.	AD
	191010	*TPM1*	15q22.2	Tropomyosin 1	N.R.	AD
